# Embodiment for Robotic Lower-Limb Exoskeletons: A Narrative Review

**DOI:** 10.1109/TNSRE.2022.3229563

**Published:** 2023-02-02

**Authors:** Rachel L. Hybart, Daniel P. Ferris

**Affiliations:** J. Crayton Pruitt Department of Biomedical Engineering, University of Florida, Gainesville, FL 32611 USA

**Keywords:** Embodiment, exoskeleton, lower-limb

## Abstract

Research on embodiment of objects external to the human body has revealed important information about how the human nervous system interacts with robotic lower limb exoskeletons. Typical robotic exoskeleton control approaches view the controllers as an external agent intending to move in coordination with the human. However, principles of embodiment suggest that the exoskeleton controller should ideally coordinate with the human such that the nervous system can adequately model the input-output dynamics of the exoskeleton controller. Measuring embodiment of exoskeletons should be a necessary step in the exoskeleton development and prototyping process. Researchers need to establish high fidelity quantitative measures of embodiment, rather than relying on current qualitative survey measures. Mobile brain imaging techniques, such as high-density electroencephalography, is likely to provide a deeper understanding of embodiment during human-machine interactions and advance exoskeleton research and development. In this review we show why future exoskeleton research should include quantitative measures of embodiment as a metric of success.

## I. INTRODUCTION

Successful real-world use of robotic exoskeletons to assist human movement will require improvements to controller designs based on an understanding of how the exoskeleton and human nervous system interact. Robotic technology has advanced enough that engineers around the world create and test wearable exoskeletons that assist human motion. The goal is to create devices with accessibility, ease of use, and functionality in mind [1]. To make these devices beneficial in real-world scenarios, they must be agile and unobtrusive. Regardless of an exoskeleton’s potential, if there are considerable barriers to its use by individuals – the device is bulky, heavy, difficult to don and doff, or too functionally specific – the costs will outweigh the benefits and the device will not be utilized by relevant populations. Likewise, controllers that do not effectively interpret the user’s intent or that create a lag between exoskeleton motion/force and user’s motion/force will not be widely adopted. Most current exoskeleton controllers rely on a combination of kinematic and kinetic sensors to estimate the user’s intent with a finite state machine using heuristic or machine learning approaches [1], [2], [3], [4]. This can work quite well for rhythmic, continuous motions with low variability (like walking on a treadmill at a constant speed), but the control approach becomes less effective for discrete tasks, high variability movements, or transitions between behaviors (like sit-to-stand, stand-to-walk, walk-to-stand, or stand-to-sit). Researchers are working towards the design of controllers that consider user intent, environmental changes, and common transition states in day-to-day activity. Some controllers use body-in-the-loop based controllers during certain states to allow for physiologically meaningful movement during certain states [5], [6].Others added environmental sensing algorithms to determine required trajectory changes based on computer vision [7], [8], [9]. Some devices have specified movement trajectories for common activities, such a sit-to-stand transitions, stair ascent, stair descent, level walking, and standing which are triggered by specific cues [10], [11]. The wide variety of control mechanisms seem to indicate a promising future for exoskeletons, yet these devices are still not widely adapted in our daily lives. This may be due to a lack of user reliance and connection to robotic exoskeletons.

One way to frame human-robot-environment interaction is within a larger discussion of embodiment. Embodiment is the acceptance of an external object as part of your own body. Consider, for instance, the use of a hammer to place a nail into wood. Carpenters become skilled with a hammer and use it better than untrained people because the use of the tool extends their peripersonal space [12], [13], [14]. Similarly, when a human loses a limb, they can adopt the prosthesis into their body schema, or how their brain views their entire body in space [15], [16], [17], [18]. The degree of embodiment for the artificial limb varies depending on the individual and the device. With time and practice, there is usually some level of embodiment of a prosthesis beyond its functionality as a tool, but it does not reach the same level of embodiment as the biological limb. Many of the current measures of embodiment are subjective in nature due to the individuality and unknowns of the source of embodiment. The current gold standard for measuring embodiment of external devices is a qualitative personal questionnaire which asks users to reflect and evaluate on a device from a functional standpoint [16], [19], [20], [21], [22], [23], [24], [25], [26], [27], [28], [29]. In the future, researchers should move away from relying on these qualitative measures and focus on the emerging quantitative measures discussed later in this review. Previous studies have shown that time, experience, and environment can all influence embodiment of devices [16], [24], [30], [31], [32], [33], [34], [35]. Embodiment can be a valuable way for engineers to assess the success of their designs, for neuroscientists to understand brain function, and for clinicians to develop better therapeutic interventions.

**Fig. 1.**
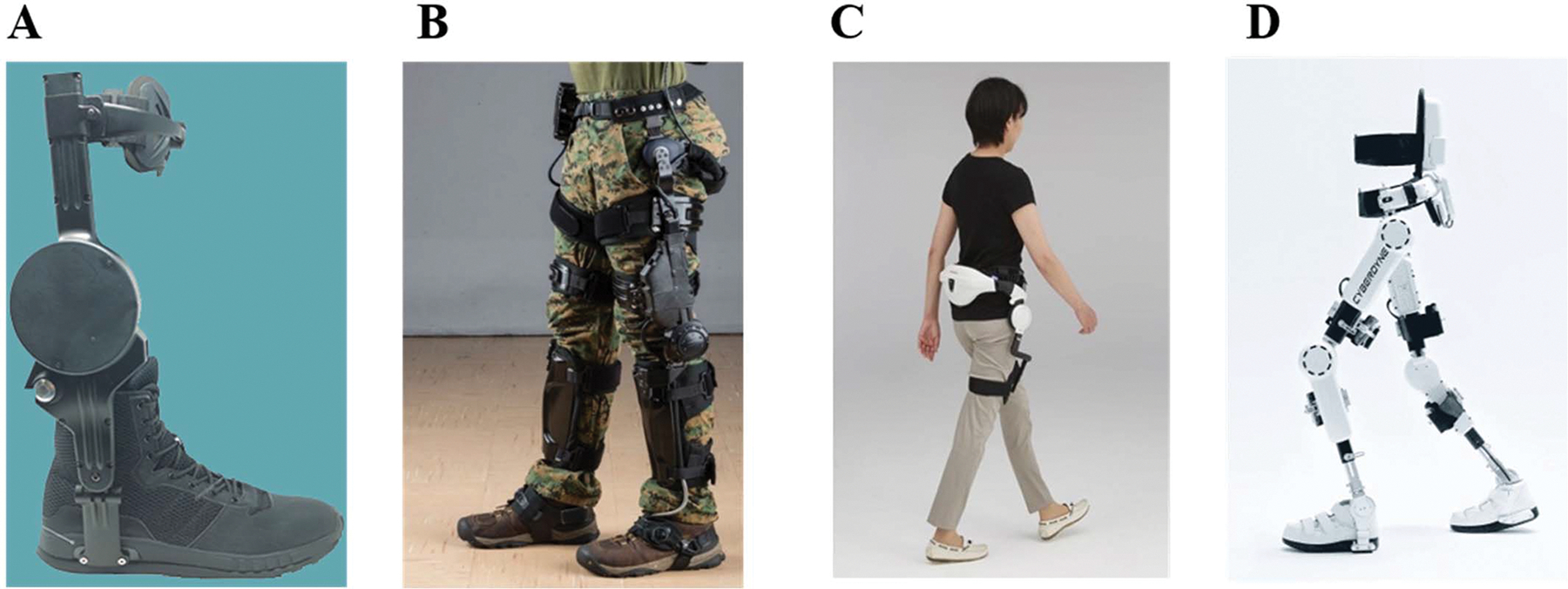
The Dephy ExoBoot ankle exoskeleton (A), the Lockheed Martin ONYX knee exoskeleton (B), and the Honda Walking Assist hip exoskeleton (C) are three examples of single joint exoskeletons that augment human performance. The Cyberdyne HAL exoskeleton (D) is a three joint rehabilitation exoskeleton.

To develop more successful robotic exoskeletons, we need to better understand how humans adopt the devices into their body schema. Robotic upper limb and lower limb exoskeletons can augment human performance in healthy individuals (Fig. 1), assist movement in individuals with neurological disabilities, or provide therapeutic treatment in individuals with neurological disabilities [36], [37], [38], [39], [40], [41]. In addition, there have been several studies using robotic exoskeletons to simulate haptic interactions in virtual reality environments, but they primarily assess immersion into the virtual world [42], [43], [44]. There are many unanswered questions fueled by the lower number of studies on embodiment of robotic exoskeletons outside of virtual reality environments. How does the quality and time of user experience impact embodiment? Does the control structure affect the embodiment of an exoskeleton? Which controller approaches produce the best embodiment? Integration of embodiment measures into the evaluation metrics of controller success may shine light on some ways researchers can improve the controller design [45]. The central thesis of this review is that the inclusion of embodiment metrics in engineering design and assessment of robotic exoskeletons will improve the success of exoskeletons to assist human movement by users in the real world.

## II. EMBODIMENT DEFINITIONS

**Fig. 2.**
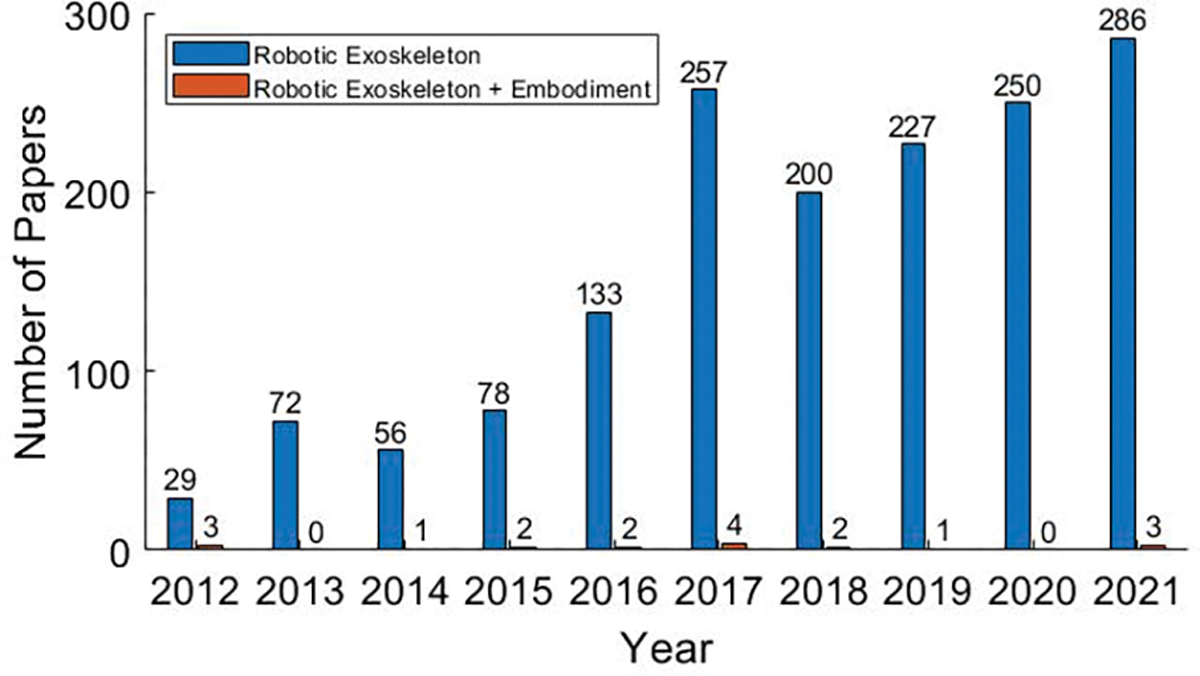
In blue are the number of publications that mention robotic exoskeletons on PubMed using the search terms: robot∗ AND exoskel∗. In orange are the publications that mention both robotic exoskeletons and one of the terms used for embodiment discussed in this paper using the search terms: (robot∗ AND exoskel∗) AND (embod∗ OR peripersonal OR “body schema” OR “body image”).

The terms embodiment, body schema, and body image describe different aspects of how the mind, body, and environment interact. Figure 2 shows the rapid growth of papers on robotic exoskeletons, and the subset of those papers which also included these key words used to describe embodiment. In the past 9 years, the number of papers referencing robotic exoskeletons has risen from 29 in 2012 to 286 in 2021. In that same time frame, the number of papers that also talked about robotic exoskeleton embodiment was a maximum of 4.

Traditionally, researchers have studied embodiment of objects by quantifying how users react to potential harm to the object, how they use the object to interact with the environment, or how they perceive it in space compared to other objects and their body [1], [46]. An important concept in those study methods are the concepts of body schema or body image. Body schema and body image refer to the body’s configuration with respect to itself as well as with respect to the surroundings, although there is some debate on the specific distinctions between the two concepts [12], [47]. For the purposes of this review, we will focus on the terms embodiment and body schema as overlapping terminology. External objects can alter one’s body schema and image such as seen with wheelchair users [30]. Over time, users include the wheelchair in their body image, which changes how they navigate the world while in their wheelchair. Both body schema and body image are subspaces of body representation and reflect how a person perceives themself. In relation to robotic exoskeletons, body schema may be affected by the addition of an exoskeleton that changes how the body can move.

Embodiment can be broken down further into different types of embodiment. Lux, et al. describe the levels of embodiment from the genetic level to the cognitive level and further to embodiment caused by social interactions [48]. This summary of the definitions is a good representation of the breadth of embodiment in our everyday lives. Although not all researchers choose to use the same terminology, there are concepts which appear repeatedly in the literature. Physical embodiment focuses on how body representation (body schema and body image) determines how a user views an external object as part of their own body. Neural embodiment reflects evidence that an object has triggered modifications in ongoing brain activity related to the object’s use or identity [49]. For example, a person experienced working with tools will demonstrate activation in sensorimotor cortex when viewing a hammer, indicating a direct association of that external object with relevant neural pathways [49], [50]. This may occur alongside motor learning, but typically takes a longer time to occur than motor learning. For instance, both experts and novice users of a tennis racket learn the motor commands to use the racket. However, only expert users embody their specific racket [32]. The opposite is seen during the Rubber Hand Illusion, where the user does not have any motor control of the rubber hand but is able to embody it. Phenomenological embodiment, in contrast, is when a user reacts to stimulation of an object in a similar way they might react to stimulation of their own body. A popular example of this is the rubber hand illusion, where physical harm to an unattached rubber hand elicits a physical response like the hand is attached to the participant [19], [51], [52], [53], [54], [55]. The different uses and definitions of embodiment in the current literature raises questions about the best way to measure embodiment, if different types of embodiment can be altered separately, and if there is one definition that is more important than the others. Later in this review we discuss some newer ways of measuring embodiment quantitatively that may provide answers to some of these questions with respect to exoskeleton embodiment.

## III. EMBODIMENT OF TOOLS AND INTERNAL MODELS

Tools have been useful for the study of embodiment as they provide a test case of how humans alter body schema in regard to external objects. Any object can be a tool if it is used to complete a specific task or set of tasks. There is evidence for embodiment of tools such as wheelchairs, hammers, and tennis rackets with prolonged use [30], [32], [49], [56], [57]. As one example, wheelchair use alters the user’s body schema so that they perceive their peripersonal space to be as wide as the chair, rather than the widest point on their body [30], [56]. Familiarity with the tool matters as well. If someone is given an unfamiliar wheelchair or tennis racket, their body schema does not change as much as when they are given the tool they typically use [32], [56], [58].

**Fig. 3.**
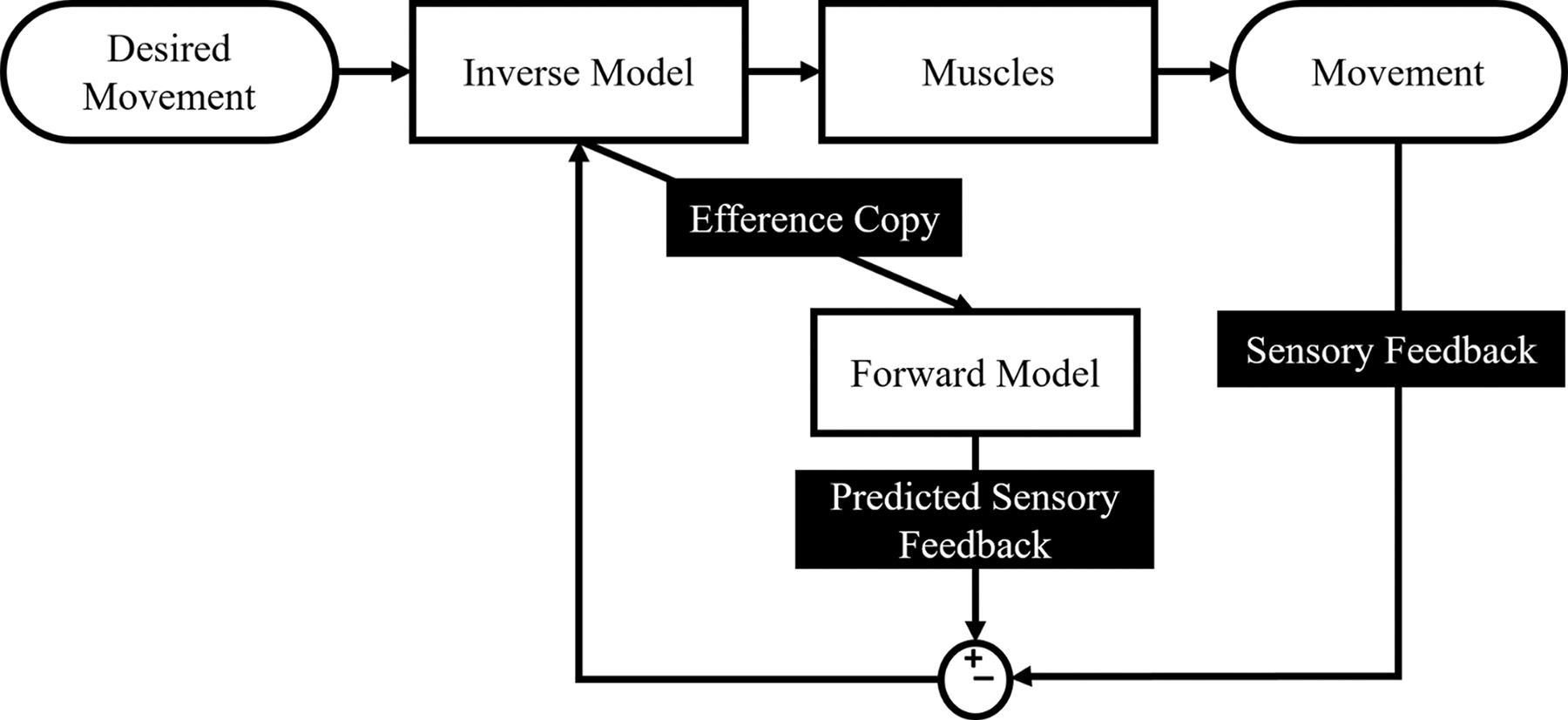
The human brain uses both an inverse and forward model of body dynamics for controlling body movements. Starting with the desired motor behavior, the nervous system calculates the appropriate efferent signals to stimulate muscles. A copy of that signal (efference copy) moves to a forward model to predict the expected sensory feedback during the movement, which is then compared to the actual sensory feedback occurring during the movement. This process is how the nervous system learns to improve control of body movements.

Efference is an important concept in understanding embodiment. Efferent signals are the signals that go from the central nervous system to the periphery. When a person wants to make a movement occur, a part of their brain containing an inverse model of body mechanics calculates the neural commands (efference) necessary to make the movement happen [59], [60], [61]. In addition to sending the efferent commands to the muscles, the nervous system also sends a copy of the efferent signals to a forward model of the body dynamics (Fig. 3). The efference copy and the forward model allow the nervous system to predict the expected sensory feedback when the movement occurs. The afferent signals are the signals sent from the peripheral nervous system to the central nervous system. These constitute the actual sensory feedback seen in Fig. 3.

By comparing the predicted sensory feedback with the actual sensory feedback, the nervous system can track errors in the control system and respond to unforeseen perturbations and improve motor precision with practice [60]. If the expected movements and actual movements consistently and repeatedly do not align, the user does not feel agency over the movement, leading to a decreased sense of embodiment [62]. Individuals with Parkinson’s Disease embody tools to a lesser degree than healthy controls, as demonstrated by spatiotemporal measures of movements with and without a stick [63]. The limited ability of individuals with Parkinson’s Disease to properly integrate sensory information and motor commands via an internal model may play a role into the reduced embodiment. As another example, a person experienced in using a wheelchair knows what forces they need to apply to move at a certain pace and in a certain direction. For the experienced user, the actual and predicted movements align well. If, instead, they were given a different wheelchair with different inertia and mechanics, it would take time to adapt efferent signals to control the new wheelchair with the same level of accuracy and precision [48]. A similar outcome is seen when advanced tennis players are given a racket they do not typically use, compared to the racket they use regularly [32]. Although they are aware of how to use the tool, the novel racket does not elicit the same responses when they move it, which leads to less embodiment of the racket.

A scientific study on efference and reafference, or a sensory response due to the subject’s own actions, that is of particular importance to robotic exoskeletons involves subjects tickling themselves. Humans generally cannot tickle themselves because they can predict the sensory feedback from the tickling, muting the response [64]. Blakemore et al. used a robotic apparatus so that subjects moved a manipulandum with one hand that moved another robotic manipulandum that tickled the second arm [65]. Even with the robotic interface, the tickling response was muted. However, when introducing a delay between the subject’s motor command the tickling sensation increased. Even with just a 100 ms delay, the subjects reported significantly greater tickle feeling than when the movement was not delayed. The increased tickle reaction occurred because the delay induced a mismatch between the forward model prediction of sensory feedback and the actual sensory feedback [65]. A similar mismatch may be seen between human and exoskeleton movement if the controller does not properly interact with the nervous system. Efference and both internal models play an important role in controlling human movement in real world environments.

Different sensory modalities contribute to the establishment and refinement of internal models. Although proprioception, how we sense movement of our body, is important in the control of our bodies, we also use vision and hearing when we learn to use tools [66], [67], [68], [69]. Using a robotic hand in grasping tasks leads to changes in activation in the sensorimotor hand area [19], [50]. In the previous example of a wheelchair user, the ability to feel the forces applied to the chair, as well as visualize movements, is important in the acceptance of the tool. If you sent a motor command to push at a specified force, but the resultant sensations were mismatched relative to that force, it may lead to a maladjustment of the internal model [59], [60], [70]. Sensory feedback provides information for the individual to know their location in space, how close they are to their desired outcome, or if the movement had unintended consequences. Comparison of expected and actual afferent feedback can lead to changes in the efferent signals as the nervous system fine tunes the internal models [60]. Achieving optimal human-machine performance requires engineers to consider how the device will affect efference copy, sensory feedback, and internal models with extended use.

## IV. DIFFERENCES IN EXOSKELETON AND PROSTHESIS EMBODIMENT

Although there has been much research on embodiment in prostheses [16], [23], [24], [71], [72], [73], robotic exoskeletons are not prostheses and therefore the research in this field cannot be necessarily applied to exoskeleton embodiment. Prostheses replace a missing part of the body while exoskeletons guide, assist, or augment intact limbs. Since prostheses replace a missing body part, movements which are not biologically plausible do not have to compete with neural signals from the intact limb like exoskeletons. For this reason, the way they are embodied may be different than an exoskeleton. When a person with an amputation uses their prosthesis of choice, it alters their peripersonal space and body schema. For example, the physical space around an individual that they perceive as reachable is different for individuals with upper-limb amputations compared to individuals without a prosthesis. When using a prosthesis of the same length as their intact limb, the space they perceive as reachable is smaller for their prosthetic limb than their intact limb and is also smaller than the perceived reachable space for individuals without an amputation [23]. Providing training sessions which involve synchronous stroking of the prosthesis and intact limb led to a correction in the misestimated limb length [74]. This may be a result of matching visual and proprioceptive feedback about the two limbs. Adding a sensory feedback system in an upper limb prosthesis led to user’s feeling as though the prosthesis was lighter in weight [75]. Perceiving a device as heavier than what is expected may lead to less embodiment of the device.

On the other hand, robotic exoskeletons intend to augment the physical capabilities of a healthy, intact individual, and provide access to constant proprioceptive feedback about the limb state as well as feedforward neural control of the limb’s motions and forces. Feedforward control of exoskeletons allows for the user’s intentions to determine the movements of the robotic device. Some examples of feedforward systems are those that use EMG based control, or exoskeletons like the ReWalk where the user tilts their pelvis and causes a specific movement from the exoskeleton. Feedforward control in prostheses typically relies on EMG from the intact portion of the limb or the contralateral limb [76], [77], [78]. Very few prostheses provide a constant updating of feedforward control and proprioceptive feedback. Researchers increased embodiment of some prostheses by including a source of neural feedback in the device [24], [74], [79], [80]. Neural feedback provides the afferent signals needed to create an accurate internal model of the device. Exoskeletons used for therapeutic rehabilitation of individuals with neurological injuries may have embodiment processes that are more like those of prosthetic limbs. Individuals with spinal cord injury or brain injury will likely not have the same level of feedback motor control or proprioceptive feedback as a neurologically intact individual. Very recently, engineers have added artificial sensory feedback to exoskeletons in hopes of improving device function, and possibly embodiment, for neurologically impaired users [30], [36], [79], [81], [82], [83] but there is not enough evidence as to the success of the approach.

In addition to the more complex controls that make exoskeleton and prosthesis embodiment different, there are also other characteristics to consider when trying to compare the two. The amount of time they are typically worn is a big difference in how users embody them. One study surveying lower limb amputees showed that they used their prosthesis for 12.47 ± 4.34 hours per day, with higher numbers related to employment status [84]. Most studies looking at adaptation to exoskeletons are short term studies where the users may only have used the device for a few hours total from start to finish. The research done on prosthesis embodiment is a good jumping off point for studying embodiment of exoskeletons. However, there are enough differences that we cannot assume the same outcomes will be seen when studying exoskeleton embodiment.

## V. ROBOTIC EXOSKELETON CONTROLS AND EMBODIMENT

**Fig. 4.**
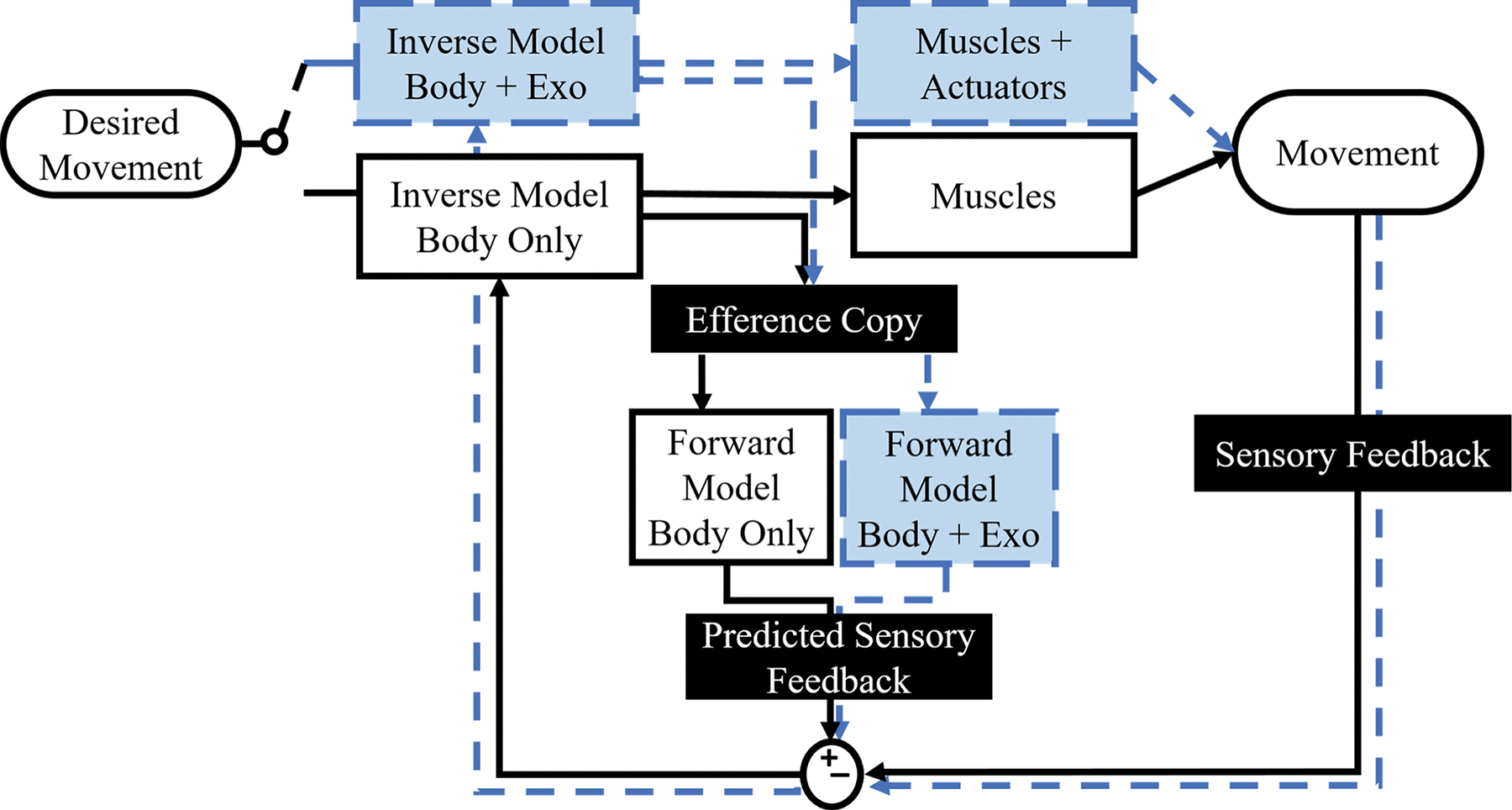
While wearing an embodied exoskeleton, a user’s internal model would switch to the track represented by the blue dashed line. This track includes an inverse and forward model which represent both the body and the exoskeleton. In addition, the muscles used to produce the movements are now working alongside the actuators of the exoskeleton. The switch after desired movements shows that only one track is followed at a time, but both are still present.

Embodiment of robotic exoskeletons will likely be dependent on whether humans can form an internal model of exoskeleton dynamics. Figure 4 provides context for how an exoskeleton may impact the neural control of movement. When a human has extensive practice with a robotic exoskeleton, the ideal scenario is that they can switch internal models from their normal biological limb to models representing the combined biological limb and exoskeleton [60], [85]. If the user does not have agency over the controller or the controller is not transparent to the user, the user would have a more difficult time developing (an) appropriate internal model(s) to reflect the altered limb dynamics with the exoskeleton [85]. There are many studies that have examined the role of internal models with respect to upper limb manipulandums and virtual sensory perturbations using virtual reality [19], [85], [86], [87], [88]. Some locomotion studies have used treadmill fixed lower limb exoskeletons to induce locomotor adaptation to mechanical perturbations [89], [90]. These studies suggest humans can learn an internal model that combines their own biological system dynamics with robotic system dynamics. Future research should examine humans using portable robotic lower limb exoskeletons to determine if practice walking transfers to other tasks such as stair climbing, cycling, or sit-to-stand movements.

Cognitive fit is another concept similar to embodiment but used in a slightly different manner. Stirling et al., define cognitive fit similarly to neural embodiment and discuss how it is important in the development of a rich internal model. The amount of cognitive load required to use an exoskeleton should not cause an increase in errors or a decrease in function in other important daily tasks [91]. If the exoskeleton controller is viewed as an external agent by the nervous system, it will create further challenges for the nervous system to adopt the exoskeleton into a common internal model of biological limb and exoskeleton dynamics.

In human movement, the nervous system uses both high-level and low-level controllers. A human may decide to stand up from sitting in a chair, walk across the room, and sit in a new chair closer to another individual. To achieve that goal, the brain sends an efferent signal to initiate the sit-to-stand and locomotion processes (i.e., a high-level control command). The spinal cord deciphers the actual neural signals that need to be sent to various muscles to generate forces within the muscle fibers to create movement. The human nervous system then initiates low-level control with sensory feedback reflexes (e.g., muscle spindle monosynaptic pathways, crossed-extensor reflex) to simplify and provide step-by-step variability of the motions.

When combining low-level control and high-level control feedback of the human body (e.g., visual and vestibular feedback to the brain about postural orientation), the controller is more robust to perturbations and movement failures [92], [93], [94], [95]. Internal models can take into account both high-level and low-level control because humans can alter feedforward strategies and reflex responses in a context-dependent manner in a given task and/or environment [85]. One example for changes in high-level control is when humans walk on a slippery, icy surface. Humans lengthen the timing of muscle synergies used for the lower resulting in more co-contraction when walking on slippery surfaces [96]. The human alters the complexity of muscle synergies used for the lower limb muscles and increase joint impedance during stance [97]. Another example of changes in low-level control comes from the seminal work by Nashner [98]. He and his colleagues demonstrated that stretch reflexes in the lower limb can vary depending on context and posture.

Current exoskeleton controllers do not incorporate these types of modifications in high-level and low-level control. Historically, exoskeleton developers have focused on high-level controllers for exoskeleton dynamics. Sensors on the exoskeleton have used kinematic or kinetic feedback to determine the general task intended by the user, and then activated the motors/actuators to assist the human motion. Increased consideration of both high- and low-level controls would enable exoskeletons to better coordinate with the human nervous system and likely increase the embodiment of devices. Increased movement variability by an exoskeleton user may make it harder for the exoskeleton to decipher the user’s intent [68], [99]. Discord between the human’s intended movement and the exoskeleton assistance/resistance would likely be registered as an error by the nervous system, requiring adjustments to existing internal models. An exoskeleton that only uses joint kinematics in a traditional state-based controller to coordinate actuator torque would miss changes in impedance and muscle activation that accompany change in terrain for example [100]. Incorporating sensors that directly measure muscle activation (electromyography or EMG) may better allow an exoskeleton to make adjustments in line with the user’s intent. Coordination of the high and low-level biological and robotic systems is necessary to create an exoskeleton that can cooperate with the user during low and high variability movements. This section aimed to briefly show how the types of robotic exoskeleton control may influence embodiment.

## VI. TECHNIQUES FOR MEASURING EMBODIMENT

Currently, most embodiment research has used questionnaires as their gold standard for determining how much a subject embodies a device. Many questionnaires include statements adapted from a rubber hand illusion questionnaire from Botvinik and Cohen [69], asking the user to rate how strongly they agree or disagree with statements about embodiment and ownership [51]. In some cases, responses from the questionnaires have been used to justify the interpretation of the quantitative physiological, or biomechanical results [16], [19], [20], [22], [23], [24], [25], [26], [27], [28], [29], [50]. In one experiment, subjects who identified their prosthesis as being more integrated into their body schema determined the reachable space around them more accurately while wearing it than subjects with less embodied prostheses [23]. Another study found that during initial training, biologically plausible joint configurations were helpful in learning how to control a robotic prosthesis, but that in later test trials, the plausibility of the joint configuration did not affect the user’s ability to correctly control the device [101]. There are other cases, however, where quantitative physiological or biomechanical measures do not show correlation with questionnaire answers [19], [29].

The most common physiological or biomechanics measures of embodiment in the literature focus on sensory perception or motor performance outcomes and are primarily indirect. Proprioceptive drift, for instance, is the measured difference between the actual location of a body part and the point where a person perceives that body part to be in space. In rubber hand experiments, proprioceptive drift has been used to determine if the participant’s perceived hand location moves closer to where the rubber hand is in space due to different perturbations [19], [102], [103]. Knowing where your body is in relation to itself and objects around you is an important defining factor in embodiment.

One category of physiological measures of embodiment includes kinematic outcomes, such as reaction time, and movement velocity. Reaction/response time is a common measure in studies of tool embodiment. Participants had faster reaction times to stimuli when they were more comfortable using a tool than less comfortable using the tool; using novel tools led to slower reaction times [32], [104]. Similarly, tasks completed with a familiar tool show increased movement velocities compared to tasks performed with an unfamiliar tool [105]. Performing a task quickly as well as accurately has been interpreted as a sign of increased embodiment of the object used in the task.

In studying embodiment of prostheses, some researchers have taken the biomechanical measure postural sway as an indicator of embodiment. Individuals with lower limb amputation that consistently use a prosthesis and show greater embodiment responses on questionnaires, demonstrate greater standing stability measured by reduced postural sway [16]. Those who use a prosthesis less frequently experience increased postural sway when wearing their prosthesis. This implies that familiarity and the quality of practice with the device matters [16]. These indirect physiological and biomechanical measures of embodiment can be helpful in understanding nervous system interactions with devices but there is still room for debate whether they are valid and robust measures of embodiment that can be translated to exoskeletons.

To say that these measures are valid measures of embodiment of an exoskeleton we will need to demonstrate that the device is valued as part of the user, rather than as an external system acting on the user. Some of the measures, such as reaction time, may show improvements while using an exoskeleton that are solely due to the intended interaction between the device and the user. For this reason, it may be necessary to combine these kinematic and biomechanical measures of physical embodiment with measures of neural and phenomenological embodiment. For example, seeing not only a faster reaction time, but also reactions to stimuli to the device related to those reaction times may validate some of the embodiment measures. In addition, to validate these physiological and biomechanical measures as measures of embodiment for exoskeleton use, we will need to understand the underlying neural basis of embodiment.

It may be more helpful for exoskeleton development to use neural measures of embodiment. There have recently been studies that have used transcranial Direct Current Stimulation (tDCS), functional Magnetic Resonance Imaging (fMRI), and Electroencephalography (EEG) to study tool or prosthesis embodiment in recent years [22], [50], [71], [79], [81], [82], [104], [108], [109]. Past EEG and fMRI studies have examined neural responses in subjects while they have been standing, sitting, or laying down. The protocols required subjects to respond to on-screen images or imagined movements [110]. Often these images are of prostheses or tools where the subject presses a button to indicate they see their own device, or they are imagining the use of a displayed tool. These studies have shown that tools used more often by the subject elicit different cortical activation patterns than tools that are unknown or less used by the subject [16], [32]. Virtual reality has been used in conjunction with EEG to show the differences in real, imagined and observed movements [111]. Perceived embodiment increased when doing or imagining the movements alongside an avatar when compared to observing the movements. This shows the importance of mobile studies to induce exoskeleton embodiment. In many instances, the EEG and fMRI studies have also included questionnaires to test for correlations between the perceived embodiment of the subject and activation in specific brain regions [23], [112]. This is an example of convergent validation, where the current accepted measures (questionnaires) are correlated to newer neural measurements (EEG and fMRI). Increased activation in the temporoparietal junction and extrastriate body area in the brain have been associated with increased embodiment [104], [113]. The temporoparietal junction is associated with processing of mental own-body transformations (viewing yourself face on vs viewing yourself in the same orientation you are in), social cognition, introspection, self-perception, and attending to unexpected stimuli. [114], [115]. The extrastriate body area is associated with perception of actions, specifically those involving nonfacial body parts [116], [117]. Combined these two areas give us a better idea of how our body is oriented and moving through space.

With the improvement of mobile EEG hardware and data processing algorithms, measuring electrocortical dynamics during active use of robotic exoskeletons becomes another possibility for quantifying embodiment. Combining high-density EEG, blind source separation techniques like independent component analysis, and subject-specific inverse head models can provide quantitative assessment of electrocortical activity in different brain regions [118], [119], [120]. These techniques and new algorithms to remove motion artifacts allow scientists to study brain dynamics during walking and running, with and without a lower limb prosthesis or robotic exoskeleton [83], [121], [122], [123], [124], [125], [126].

EEG and other measurement modalities, such as functional near infrared spectroscopy (fNIRS), provide insight into brain function on topics adjacent to the study of embodiment that suggests it may be useful for measuring embodiment. One study used fNIRS to show connectivity between the supplementary motor area and the medial prefrontal cortex was more prominent in early adaptation compared to late adaptation to a passive exoskeleton. Changes in connectivity from early to late adaptation may allow researchers to parse out differences in brain activity related to adaptation and those that may be related to embodiment [127]. Another fNIRS study looked at differences between passive and active exoskeleton assistance. They found increased activation in the parietal cortex which is often associated with motor performance, with the subcortical inferior parietal region being associated with embodiment [115], [128], [129], [130]. One EEG study showed it is possible to discern if someone is feeling a positive or negative emotion (emotional valence) with EEG [131], [132], which may provide important insight into understanding a person’s emotions towards an object. EEG measurements in human subjects using a robotic exoskeleton could reveal emotional valence towards the exoskeleton, providing insight into how the user perceives the device. This possibility is supported by evidence that EEG can detect differences in how a user perceives their environment. Gramann et. al found that there were differences in cortical activation patterns when a participant views the surroundings with respect to themself (egocentric) compared to participants that view the surroundings with respect to one another (allocentric) [133]. This relates to the phenomenological definition of embodiment where the embodiment of the exoskeleton results in reflexive reactions to stimuli to the device similar to if the person’s own body where stimulated. The types of stimuli used are often those that cause a fear of harm, such as a hammer strike to a rubber hand after eliciting embodiment. These types of responses often hold an emotional response form the user, and so being able to measure these types of responses to stimuli to the exoskeleton through EEG may provide helpful insights into the neural correlates of embodiment. EEG has been used to detect real vs. avatar based errors in virtual reality environments, proving the ability to discern types of errors using event related potentials [59]. Based on these studies, it may be possible to quantify embodiment with EEG in real world situations without the use of questionnaires.

## VII. FUTURE DIRECTIONS

**Fig. 5.**
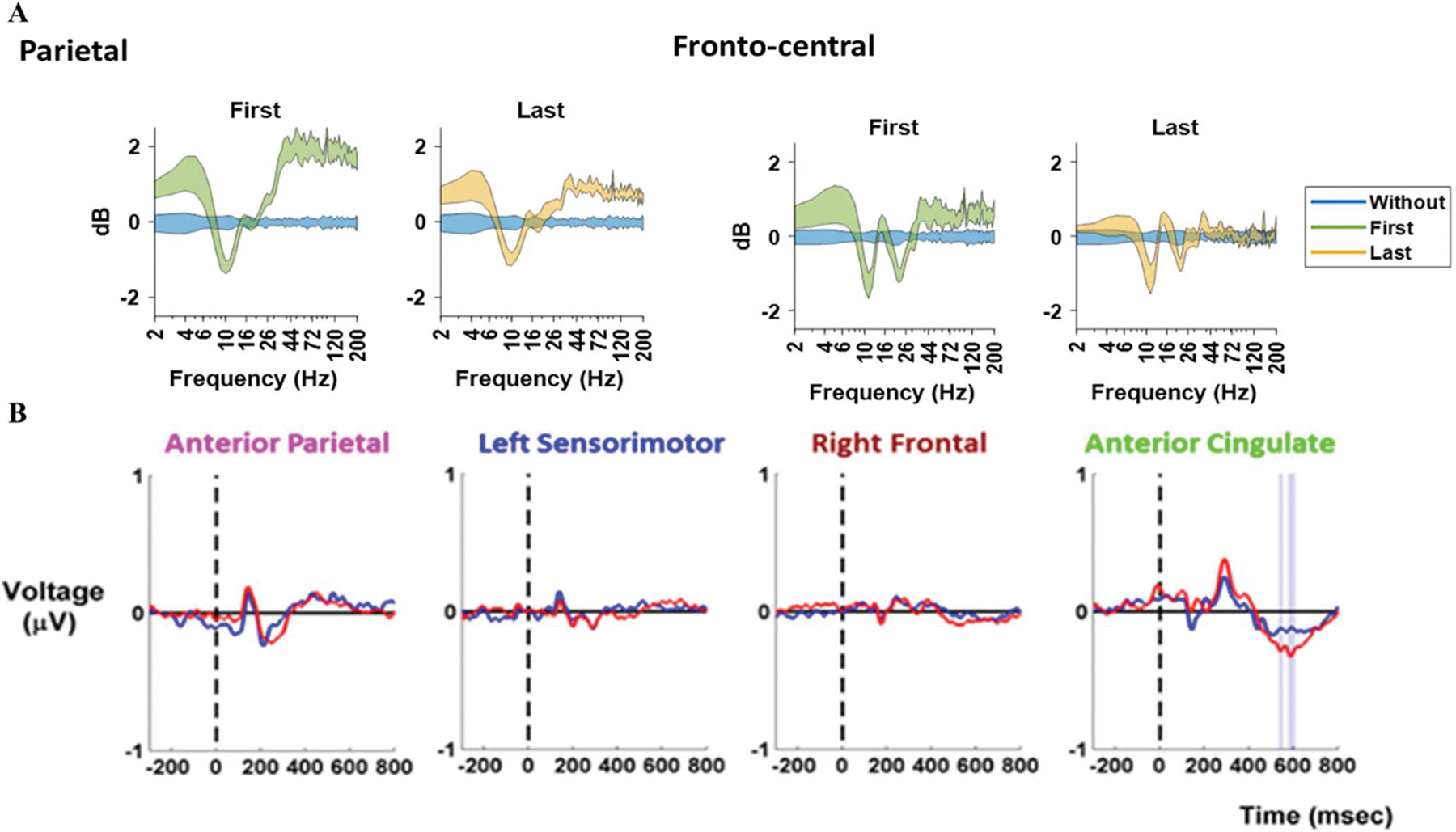
(A) Average Event Related Spectral Perturbations from fronto-central and parietal brain regions during walking with and without a dummy prosthetic leg adapted from Kooiman et al. [106]. Baseline walking condition (blue), first time walking with the dummy prosthesis (green), and final time walking with the dummy prosthesis (orange) are shown. (B) Event related activity in the anterior parietal, left sensorimotor, right frontal, and anterior cingulate cortices during walking on a balance beam without VR (red) and with VR (blue). 0 ms marks the time a tone was heard by the participant. The shaded area, seen only in the anterior cingulate, indicates a significant difference between conditions adapted from Peterson et al. [107].

As mentioned in Section II there are several definitions of embodiment. There are improvements to be made in the quantification of embodiment within each of these definitions. For all definitions, longitudinal studies may provide insight into how these different definitions/types of embodiment are related to one another. This can help to answer questions like, do users experience all types of embodiment at once? Or does one type of embodiment need to occur for the others to be experienced? To better understand neural embodiment and cognitive fit of exoskeletons, researchers need to complete more mobile studies in the real world using modalities such as EEG and fNIRS [91], [134]. Fig. 5 shows two studies that support researchers can use EEG to study differences in baseline and experimental conditions adjacent to embodiment and exoskeletons. Fig. 5 A shows average Event Related Spectral Perturbations (ERSPs) from the fronto-central and parietal regions during initial and final walking with a prosthetic emulator compared to baseline walking [106]. This study shows that changes over time and when compared to normal walking are seen in brain regions that are related to movement planning and error monitoring. Both of which will be key in determining adaptation and embodiment to exoskeletons. The changes in the ERSP in the motor cortex are related to motor learning, and the changes seen in the anterior cingulate may be related to both motor learning and embodiment. Further studies should determine the validity of embodiment metrics in terms of both convergent and discriminant validity. This will require using multiple measures across multiple time scales to determine if there are correlations between current and proposed measures of embodiment and motor learning.Future studies should look at neural measurements in these areas while completing tasks that are more in line with previous embodiment research. For instance, completing pre and post adaptation biomechanical tests for reaction time, movement velocity and postural sway while measuring EEG or fNIRS may provide more insight into how the changes in ERSPs relate to changes in previously studied embodiment measurements. Fig. 5 B shows event related activity comparing walking on a balance beam with (blue) and without (red) virtual reality [107]. Significant differences in these conditions are seen in the anterior cingulate cortex. The anterior cingulate cortex is key in error monitoring and adjustment necessary to incorporate new devices and tools into a usable internal model. The importance of these studies is justified by previous stationary embodiments studies where the temporoparietal junction was implicated in embodiment [113], [114], [115], and mobile studies where the posterior parietal cortex was associated with movement planning [124], [135].

Currently, phenomenological embodiment is tested by inducing embodiment and then applying a stimulus to the tool or device the user has embodied to quantity their reaction. Phenomenological embodiment should be tested outside of a laboratory setting. Users should freely explore a space with an exoskeleton in a way that is more natural, so that when a perturbation or stimuli is applied to the device, the user is able to react in a way that is typical of day-to-day behavior. In addition, we suspect that the measures of embodiment will vary depending on the type of controller the exoskeleton implements. With more intuitive controllers leading to faster adaptation, increased embodiment, and clear changes from the beginning of training in both neural and phenomenological embodiment. Understanding how users embody exoskeletons is an important step towards improving the design and control of exoskeletons.

## VIII. CONCLUSION

Embodiment is an understudied but important aspect of human adaptation to exoskeletons. Qualitative and quantitative measures of embodiment have been used to assess integration of prostheses and tools into the body schema of users indicate that embodiment of robotic movement devices improves the functionality of these devices. To achieve widespread acceptance of exoskeletons in everyday activities, it will be necessary for researchers to include embodiment measures in their evaluation of new devices. Combining mobile EEG or fNIRS with kinematic and biomechanical measurements (such as reaction time and proprioceptive drift) has the potential to provide new insight into exoskeleton embodiment and increasing user acceptance of new devices.
